# Influence of intranasal and carotid cooling on cerebral temperature balance and oxygenation

**DOI:** 10.3389/fphys.2014.00079

**Published:** 2014-02-27

**Authors:** Lars Nybo, Michael Wanscher, Niels H. Secher

**Affiliations:** ^1^Department of Nutrition, Exercise and Sport Sciences, University of CopenhagenCopenhagen, Denmark; ^2^Department of Cardiothoracic Anaesthesia, Copenhagen University Hospital RigshospitaletCopenhagen, Denmark; ^3^Copenhagen Muscle Research Center, Department of Anaesthesia, Rigshospitalet, University of CopenhagenCopenhagen, Denmark

**Keywords:** balloon catheter, brain temperature, cerebral oxygenation, cooling, hypothermia

## Abstract

The present study evaluated the influence of intranasal cooling with balloon catheters, increased nasal ventilation, or percutaneous cooling of the carotid arteries on cerebral temperature balance and oxygenation in six healthy male subjects. Aortic arch and internal jugular venous blood temperatures were measured to assess the cerebral heat balance and corresponding paired blood samples were obtained to evaluate cerebral metabolism and oxygenation at rest, following 60 min of intranasal cooling, 5 min of nasal ventilation, and 15 min with carotid cooling. Intranasal cooling induced a parallel drop in jugular venous and arterial blood temperatures by 0.30 ± 0.08°C (mean ± SD), whereas nasal ventilation and carotid cooling failed to lower the jugular venous blood temperature. The magnitude of the arterio-venous temperature difference across the brain remained unchanged at −0.33 ± 0.05°C following intranasal and carotid cooling, but increased to −0.44 ± 0.11°C (*P* < 0.05) following nasal ventilation. Calculated cerebral capillary oxygen tension was 43 ± 3 mmHg at rest and remained unchanged during intranasal and carotid cooling, but decreased to 38 ± 2 mmHg (*P* < 0.05) following increased nasal ventilation. In conclusion, percutaneous cooling of the carotid arteries and intranasal cooling with balloon catheters are insufficient to influence cerebral oxygenation in normothermic subjects as the cooling rate is only 0.3°C per hour and neither intranasal nor carotid cooling is capable of inducing selective brain cooling.

## Introduction

Cooling of the brain is of relevance for preventing cerebral ischemia during anesthesia and after cardiac arrest hypothermia may improve neurological outcome and even survival (Hoesch and Geocadin, 2007; Holzer, 2008, 2013; Lay and Badjatia, 2010; Harris et al., 2012). Cerebral cooling can be induced by global lowering of the body temperature as arterial blood will gradually lower brain temperature (Nybo et al., 2002; Holzer, 2008). However, methods have been developed in attempt to selectively cool the brain, i.e., without affecting other parts of the body (for review see Harris et al., 2012) in order to attenuate the risk of, e.g., pneumonia and sepsis (Geurts et al., 2014). Selective-brain cooling is defined as a lowering of the average brain temperature to below that of arterial blood as observed in several animal species including mammals with a carotid rete (Jessen, 2001). Whether humans, despite the lack of a carotid rete, have the ability to selectively cool their brain remains controversial (Brengelmann, 1993; Cabanac, 1993; White et al., 2010), but is probably unlikely under normal circumstances (Nybo et al., 2002; Maloney et al., 2007; Nybo and Secher, 2011). Yet, various intranasal cooling techniques have been developed (Harris et al., 2012) and Covaciu et al. (2011) report from a magnetic resonance (MR) spectroscopic-based evaluation of cerebral temperature that intranasal cooling with balloon catheter induced a rapid and substantial lowering of the brain temperature. Springborg et al. (2013) also find that intranasal cooling lowers cerebral temperature in hyperthermic brain-injured patients. However, in contrast to the observations presented by Covaciu et al. (2011), Springborg et al. (2013) report brain cooling to take place in parallel with normalization of the core temperature in their hyperthermic patients. Hence, it remains unclear whether intranasal cooling can induce selective brain cooling and to what extend it lowers brain temperature in normothermic subjects and thereby influences the cerebral metabolic rate and its oxygenation.

Cooling of the carotid arteries, either by percutaneous cooling of the neck or through augmented heat release from upper respiratory airway induced by increased ventilation could influence the temperature of arterial blood entering the brain (Rasch et al., 1991). As demonstrated during exercise, hyperpnea lowers tissue temperature adjacent to the carotid arteries and could thereby narrow the arterio-venous temperature difference across the brain (Nybo et al., 2002). However, in resting subjects the effect of increased nasal ventilation on brain temperature is not clear. Therefore, the present study was conducted to evaluate the effects of intranasal cooling, percutaneous cooling of the carotid arteries, and nasal ventilation on cerebral temperature balance and oxygenation.

## Materials and methods

Six healthy male subjects at a mean age of 30 ± 4 years (± SD), height of 185 ± 5 cm and weight 79 ± 8 kg participated in the study as approved by the local ethics committee (protocol H-4-2010-081) and conducted in accordance to the Declaration of Helsinki.

The subjects arrived at the laboratory in the morning ~1 h before the start of the experiment and were instrumented with thermocouples to record forehead, cheek, and neck (over the left carotid artery) skin temperature (Ellab, Copenhagen, Denmark) an ultra sound transcranial Doppler probe, and a heart rate (HR) monitor. Then, the subjects were provided with a 18 G catheter (32 mm; BD A/S, Denmark) in the brachial artery of the non-dominant arm and, under local anesthesia, a 5 F Swan-Ganz catheter (Edwards; USA) was placed in the right internal jugular vein and advanced to the bulb of the vein. A thermocouple (model MAC-07170-A, Ellab) was inserted via the arterial catheter and advanced to the aortic arch to record arterial temperature (Nybo et al., 2002), while the internal jugular venous blood temperature was obtained from the temperature sensor positioned at the bulb of the internal jugular vein. Furthermore, intra-nasal temperature was measured 1 cm into the nostrils with a thin thermocouple (MHA model, Ellab, Copenhagen, Denmark) inserted for 2 min (and until the measure was stable) with the tip/electrode directed outward; i.e., during the nasal cooling period into the tissue and away from the balloon catheter.

Simultaneous blood samples were obtained from the two catheters at baseline (after 45 min of supine rest), following 1 h of intra-nasal cooling, following 15 min of carotid cooling, and at the end of a 5 min period with increased nasal ventilation during which the subjects were instructed to double their ventilation and inhale exclusively through the nose and exhale through the mouth. All blood samples were immediately analyzed for PO_2_, PCO_2_, oxygen saturation, hemoglobin, glucose, and lactate (ABL 800, Radiometer, Copenhagen, Denmark). Cerebral arterio-venous differences for oxygen (a-vDO_2_), glucose (a-vD_glucose_), and lactate (a-vD_lactate_) were determined on basis of paired blood samples. Furthermore, changes in mean cerebral capillary oxygen saturation and capillary oxygen tension were calculated according to Rasmussen et al. (2006) with the assumption that oxygen extraction rises linearly with distance as blood traverses the capillary network from the arterial to venous end, and the average capillary bed satisfy coequal amounts of brain tissue.

Middle cerebral artery mean blood velocity (MCA V_mean_) was monitored by transcranial Doppler (Transcan, EME, Überlingen, Germany) to estimate changes in cerebral blood flow (CBF). The best signal–noise ratio at the proximal part of the MCA was selected and the vessel was insonated at a depth of ~50 mm with the probe secured with a headband. MCA V_mean_ was computed from the integral of the maximum frequency Doppler shifts over each heartbeat and the average from 2 min was determined for rest, nasal cooling, carotid cooling, and nasal ventilation. It was assumed that the diameter of the insonated vessel remains unchanged across the evaluated conditions. Serrador et al. (2000) found no variation in vessel diameter with changes in P_a_CO_2_ and it appears that the CBF is regulated distal to the proximal part of MCA, although some effect of P_a_CO_2_ on vessel diameter cannot be excluded (Valdueza et al., 1999). However, changes in MCA velocity correlate with those in ^133^Xenon determined CBF (Jørgensen, 1995) and we estimated changes in CBF from the percentage change in MCA V_mean_.

### Cooling interventions

The intra-nasal cooling was applied via two single-use intranasal balloon catheters (QuickCool Disposable Balloon Catheter, QuickCool AB, Lund, Sweden) perfused with cold isotonic saline from a heat exchanger in a closed circuit system (ComVic, QuickCool AB, Lund, Sweden). The pressure in the balloons was maintained between 20 and 30 mmHg and flow exchange was set to 200 ml per min with the temperature in the heat exchanger at 1°C. The cooling period was 1 h and all six subjects tolerated and completed the entire period without any adverse effects.

Nasal ventilation was initiated 2 min after removal of the balloon catheters in attempt to increase cooling of the upper respiratory airways while the tissue in the nasal sinuses was low.

The subject was instructed to inhale forcefully through the nose and exhale via the mouth for 5 min to maximize the potential cooling effects and that was accomplished by all subjects. Blood samples were drawn and temperatures registered during the last 30 s of the 5 min intervention. Following the nasal ventilation test, the subject rested for at least 45 min or until arterial blood temperature and P_a_CO_2_ had returned to baseline values. Thereafter 15 min of carotid cooling was applied by placing ice packets on both sides of the neck (~10 cm long and 5 cm thick plastic bag filled with crushes ice and wrapped in a thin piece of fabric to avoid freezing the skin). One subject however tolerated this intervention for only 10 min, but developed a similar drop in skin temperature as the other five subjects.

### Statistical analysis

Values are presented as mean ± SD unless otherwise indicated. Changes over time, i.e., during the period with nasal cooling or across conditions (baseline, intra-nasal cooling, nasal ventilation and carotid cooling) were evaluated with repeated One-Way ANOVA and the significance level was set at *P* < 0.05. In case of a significant difference across conditions, a Tukey *post-hoc* test with Bonferroni correction was used to identify differences.

## Results

**Figure 1:**
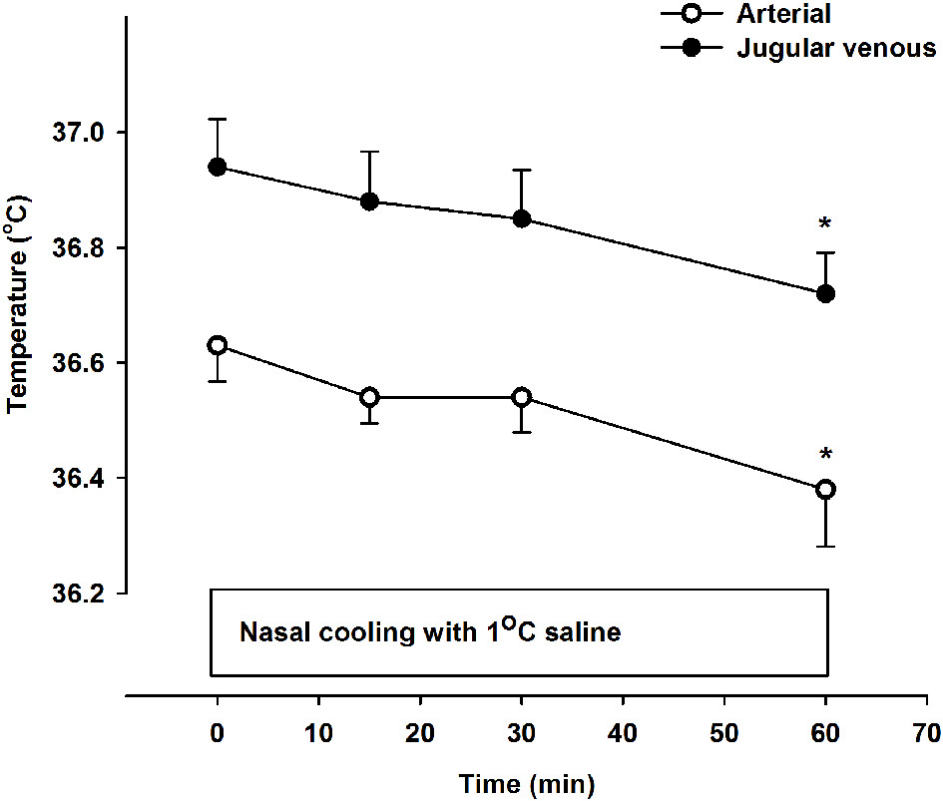
**Arterial (open symbols) and jugular venous blood temperature (filled symbols) at rest (0 min) and during 60 min of nasal cooling with 1°C saline circulated through the nasal catheters at a flow rate of 200 ml/min**. ^*^Signifies that the value is lower compared to corresponding value at 0 min (*P* < 0.05). The jugular venous blood temperature was significantly higher than that of the arterial blood at all time points (*P* < 0.001).

**Figure 2:**
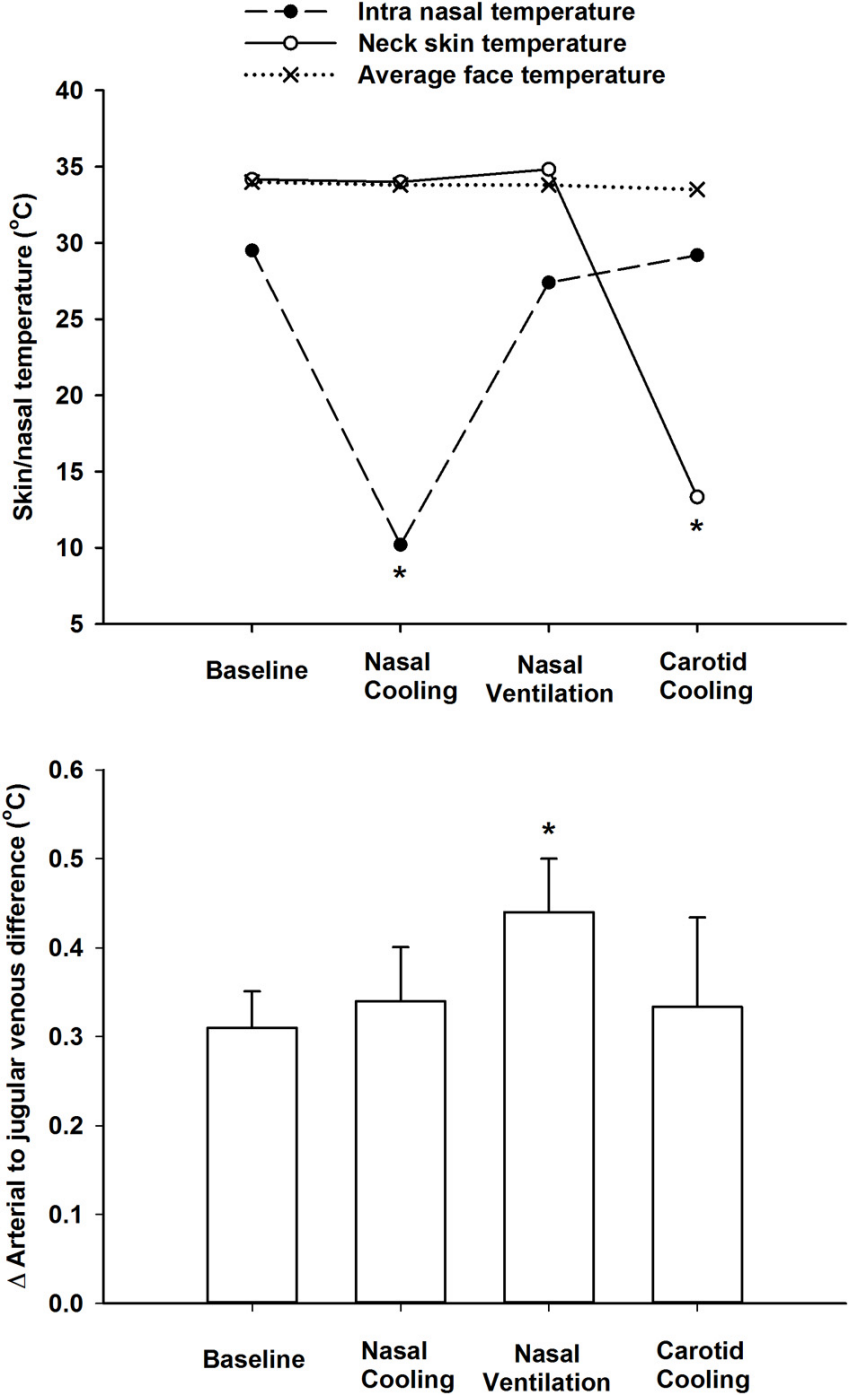
**Top panel**. Average face skin (mean of cheek and forehead), neck skin over the carotid arteries, and intra-nasal temperatures at rest (baseline), during intranasal cooling, increased nasal ventilation and carotid cooling. **Lower panel** shows the delta difference between arterial (aortic arch) and internal jugular venous blood temperatures at rest during intranasal cooling, increased nasal ventilation and carotid cooling. ^*^Indicates that the value is significantly different from corresponding value at rest (*P* < 0.05).

There was a small but significant decline in internal jugular venous blood temperature during the 1 h period with nasal cooling (Figure 1) occurring in parallel with the drop in body temperature as the arterio-venous temperature difference across the brain remained unchanged at −0.33 ± 0.05°C. Furthermore, the arterio-venous temperature difference across the brain was not changed during carotid cooling, whereas it was widened to −0.44 ± 0.11 °C following the period with nasal ventilation. Thus, the jugular venous blood temperature remained in the range 0.3–0.44°C above that of the arterial blood despite marked reductions in intranasal, neck, and face skin temperatures as illustrated in Figure 2.

**Figure 3:**
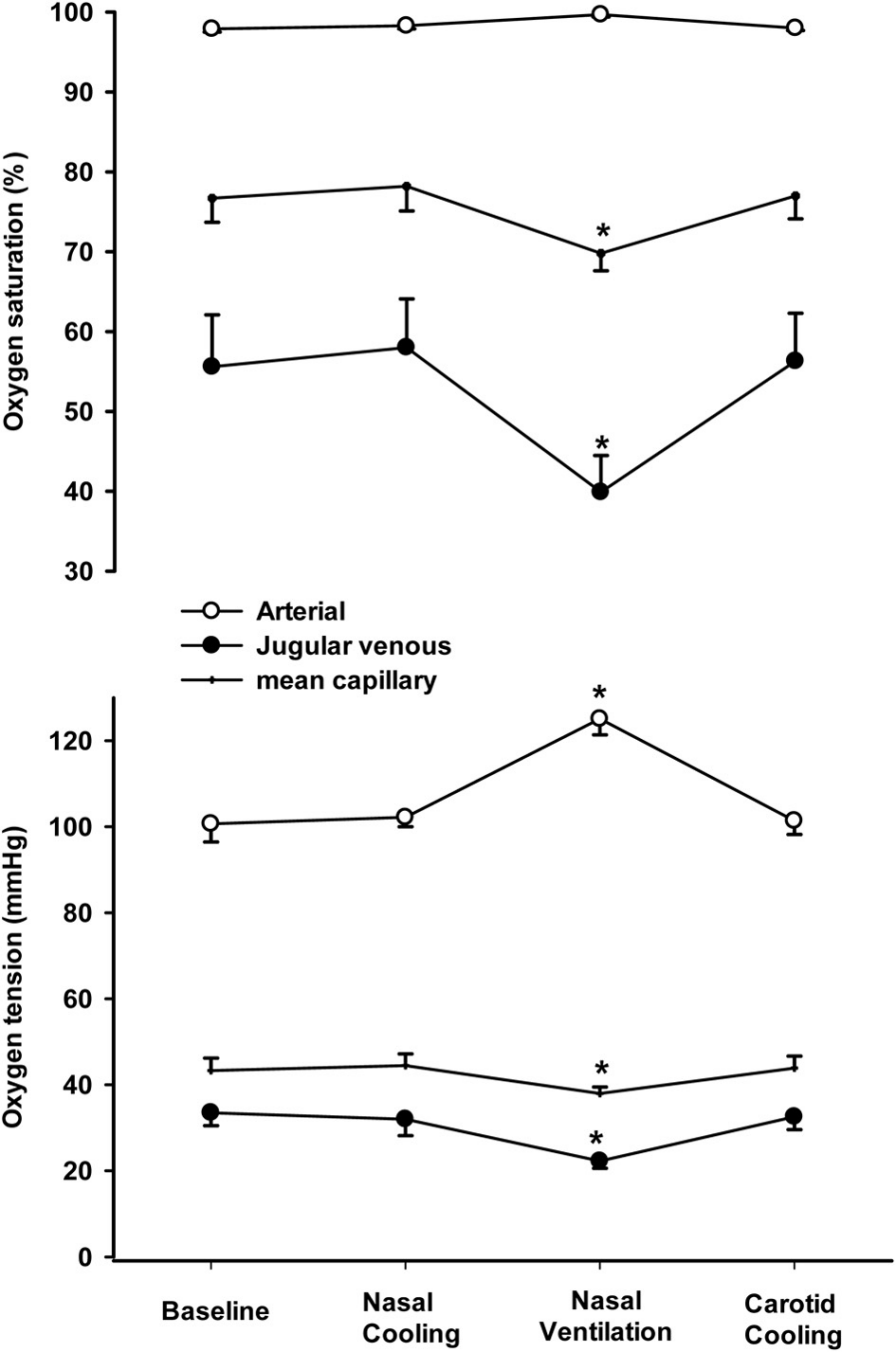
**Arterial, jugular venous and mean capillary oxygen tension (lower panel) and saturation (top panel) at rest (baseline), following 1 h of intranasal cooling, after 5 min of increased nasal ventilation and 15 min of carotid cooling**. ^*^Indicates that the value is different from corresponding value at rest (*P* < 0.05).

MCA V_mean_ remained unchanged (within 2% of baseline values) during intranasal and carotid cooling, whereas it declined to 45 ± 7% of the baseline value at the end of the 5 min period with nasal ventilation. Accordingly, PaCO_2_ was similar at baseline (39.2 ± 0.7 mmHg) during intra-nasal (39.1 ± 0.9 mmHg) and carotid cooling (39.5 ± 0.9 mmHg), but declined to 20.9 ± 3.2 mmHg following 5 min of nasal ventilation. Furthermore, PaO_2_ and saturation were similar at baseline, following intranasal and carotid cooling (average PaO_2_ ~100 mmHg and saturation ~97.5%), but increased to 125.1 ± 3.7 mmHg and 99.6 ± 0.2% following the 5 min period with increased nasal ventilation (Figure 3). However, a-v DO_2_ increased from 83.5 ± 5.5 ml·l^−1^ at rest to 119.1 ± 7.0 ml·l^−1^ following the nasal ventilation period and the jugular venous and mean cerebral capillary oxygen tension were lowered by ~10 and 5 mmHg, respectively. In contrast, a-v DO_2_, jugular venous PO_2_ and mean cerebral capillary oxygen tension remained unchanged following intranasal and carotid cooling (Figure 3, lower panel). Also, a-vD_glucose_ was similar at rest, following intranasal, and carotid cooling with an average of 0.55 ± 0.08 mmol·l^−1^ and the cerebral release of lactate remained low with an a-vD_lactate_ of −0.05 ± 0.03 mmol·l^−1^. In contrast, a-vD_glucose_ increased to 1.04 ± 0.16 mmol·l^−1^ and a-vD_lactate_ was widened to −0.20 ± 0.07 mmol·l^−1^ following the period with nasal ventilation.

## Discussion

The present study shows that intranasal cooling may lower the cerebral venous blood temperature indicating that the technique is capable of affecting the average brain tissue temperature. However, the cooling was modest and related to body core cooling and intranasal cooling did not selectively cool the brain. In the studied healthy normothermic subjects, the cooling rate was 0.3°C per hour and insufficient to influence cerebral oxygenation following 60 min. In addition, neither nasal ventilation nor carotid cooling was capable of providing a significant lowering of the cerebral venous blood temperature and also failed to increase cerebral capillary oxygenation. On the basis of these observations we conclude that intranasal cooling with balloon catheters is not recommendable for rapid cooling of the brain or to improve cerebral oxygenation and it does not selectively cool the brain.

The cerebral cooling rate achieved with intranasal cooling was comparable to that reported by other head cooling device applications in normothermic subjects (Koehn et al., 2012; Poli et al., 2013), but the rate was somewhat lower than that obtained in hyperthermic (Abou-Chebl et al., 2011; Springborg et al., 2013) and normothermic stroke patients (Poli et al., 2014) with intranasal cooling. Also the cerebral cooling was substantially slower than the rates obtained in normothermic patients with cooling induced via veno-venous extracorporeal circulation which may lower the arterial blood and brain temperatures in parallel with cooling rates of ~3.5°C per hour (Piepgras et al., 1998) or even faster (Testori et al., 2013). That the cerebral cooling induced via intranasal cooling relates to general body core cooling and not to selective brain cooling is in accordance with the observations by Springborg et al. (2013) in hyperthermic brain-injured comatic patients. In contrast, Covaciu et al. (2011) report that the balloon catheter method we used with intra-nasal cooling induced a drop in brain temperature which exceeded the decline in rectal temperature indicating that the method could introduce semi-selective cooling of the brain. However, while we tracked changes in cerebral temperature by a continuous measure of the cerebral venous blood temperature and Springborg et al. (2013) measured brain temperature directly, Covaciu et al. (2011) evaluated brain temperature changes using MR spectroscopic imaging and they tracked changes in body core temperature by measures of rectal temperature that responds only slowly to changes in core temperature (Nielsen and Nielsen, 1962). Thus, methodological differences may explain the discrepancy between observations.

The Q_10_ effect on the cerebral metabolic rate for oxygen is ~2 (Klementavicius et al., 1996; Nybo et al., 2002). Considering, the modest cooling of ~0.3 degrees achieved in the present study and the unchanged CBF and P_a_O_2_ it seems reasonable that the cerebral oxygenation remained unchanged following the 60 min period with intranasal cooling or following the carotid cooling. The increased nasal ventilation immediately following the nasal cooling was introduced in attempt to increase the heat release from the upper respiratory track that has been hypothesized to influence brain temperature (Rasch et al., 1991; Mariak et al., 1999). However, increased heat release from the brain was presumably not established following nasal ventilation as MCA V_mean_ declined by more than 50% indicating a marked lowering of CBF in the hypocapnic condition. Heat release from the brain is determined by the product of the arterio-venous temperature difference across the brain, CBF and the specific heat capacity of blood (Nybo et al., 2002) and although the present data do not allow for calculation of the cerebral heat balance, a marked lowering of CBF would outweigh the increased blood temperature difference across the brain following the period with nasal hyperventilation. Furthermore, the hyperventilation-induced hypocapnia was associated with increased lactate release from the brain indicating that reduced CBF and consequently lower cerebral oxygen delivery may have compromised aerobic metabolism and we observed that the mean capillary and venous oxygen tension were reduced following the period with nasal ventilation. All subjects tolerated the 5 min period with nasal ventilation without reporting signs of dizziness, but the hypocapnic level and marked reduction in MCA V_mean_ indicate that they were close to levels that may lead to syncope (Immink et al., in press). If normal alveolar ventilation and consequently also P_a_CO_2_ had been maintained during the nasal ventilation, it is almost certain that cerebral oxygenation had also remained stable, however, we asked the subjects to inhale forcefully through the nose to maximize the potential cooling effects this could have on the upper respiratory tract. Therefore, hyperventilation-induced hypocapnia was introduced and the associated lowering of cerebral oxygen delivery was expected (Kety and Schmidt, 1948) during this part of the experiment targeted at optimizing cooling and not at enhancing the cerebral oxygenation.

## Concluding remarks

Intranasal cooling with balloon catheters was insufficient to influence the cerebral oxygenation in awake, healthy subjects. The cooling procedure lowered the temperature within the nasal cavity by ~20°C but the effect on the cerebral temperature was modest with an estimated cooling rate of 0.3°C per hour and the cooling was not selective for the brain as the arterio-venous temperature difference across the brain remained unchanged. In addition, neither nasal ventilation nor bilateral percutaneous cooling of the carotid arteries was capable of providing significant lowering of the cerebral venous blood temperature and these methods also failed to increase cerebral capillary oxygenation.

## Author contributions

Lars Nybo designing, planning and conducting the experiments, analysis of data and writing the manuscript; Michael Wanscher designing, planning and conducting the experiments and contributing to the manuscript; Niels H. Secher designing, planning and conducting the experiments and contributing to the manuscript

### Conflict of interest statement

The authors declare that the research was conducted in the absence of any commercial or financial relationships that could be construed as a potential conflict of interest.
